# Supplementary figures and images for: VPS38/UVRAG and ATG14, the variant regulatory subunits of the ATG6/Beclin1-PI3K complexes, are crucial for the biogenesis of the yolk organelles and are transcriptionally regulated in the oocytes of the vector Rhodnius prolixus
Source: PLoS Negl Trop Dis. 2021 Sep 7;15(9):e0009760. doi: 10.1371/journal.pntd.0009760 (PMC8448300; doi:10.1371/journal.pntd.0009760)

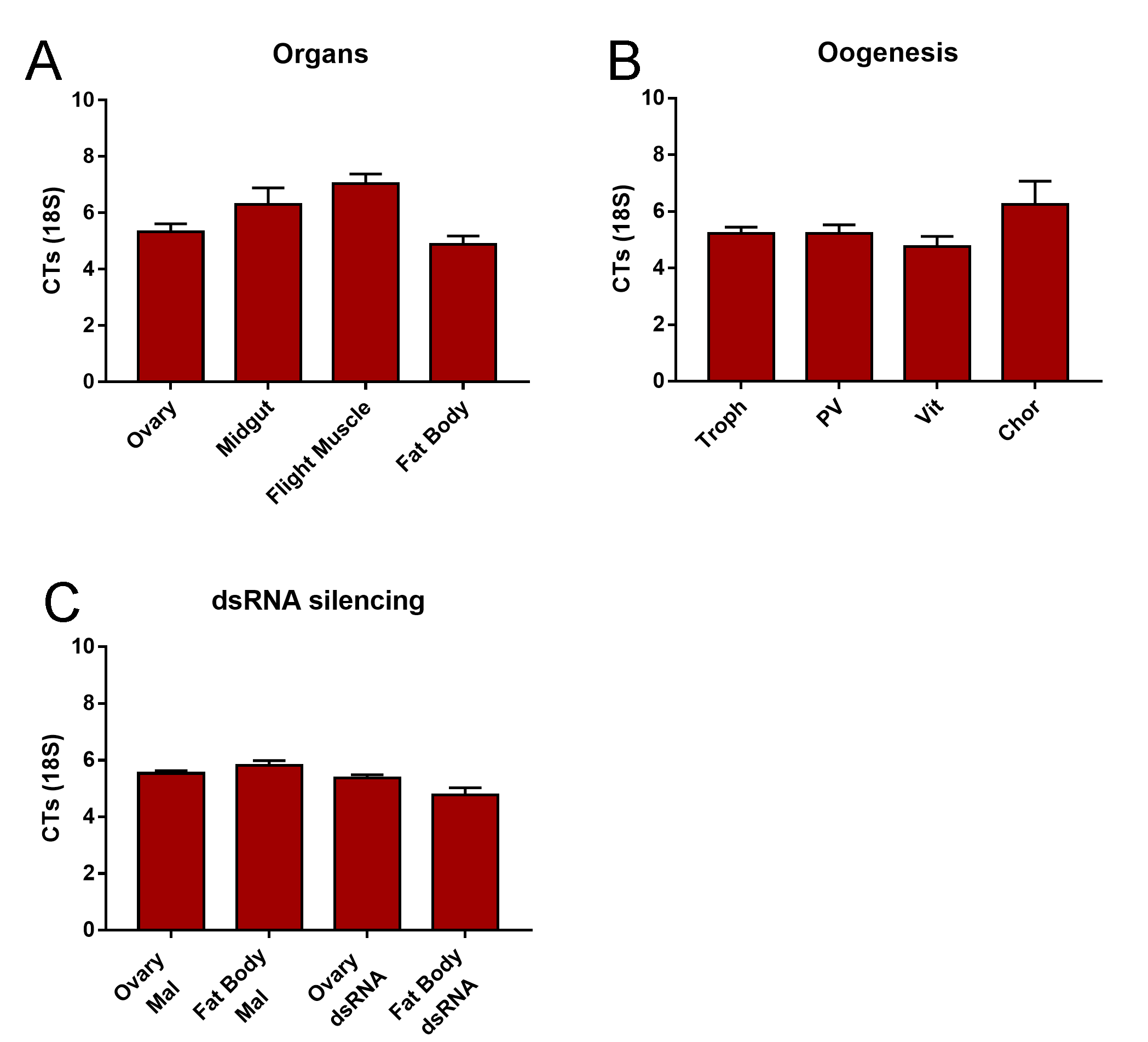

Supplement: S1 Fig — 18s Cts obtained from different samples and conditions. (A) RNA extracted from the ovary, midgut, fat body and flight muscle. (B) RNA extracted from the different parts of the ovariole (troph, tropharium; PV, previtellogenic follicle; Vit, Vitellogenic follicle; Chor, chorionated oocyte). (C) RNA extracted from the ovary and fat body of control (dsMal) and silenced insects. All samples were dissected 7 days after the blood meal. (TIF) [file pntd.0009760.s001.tif]

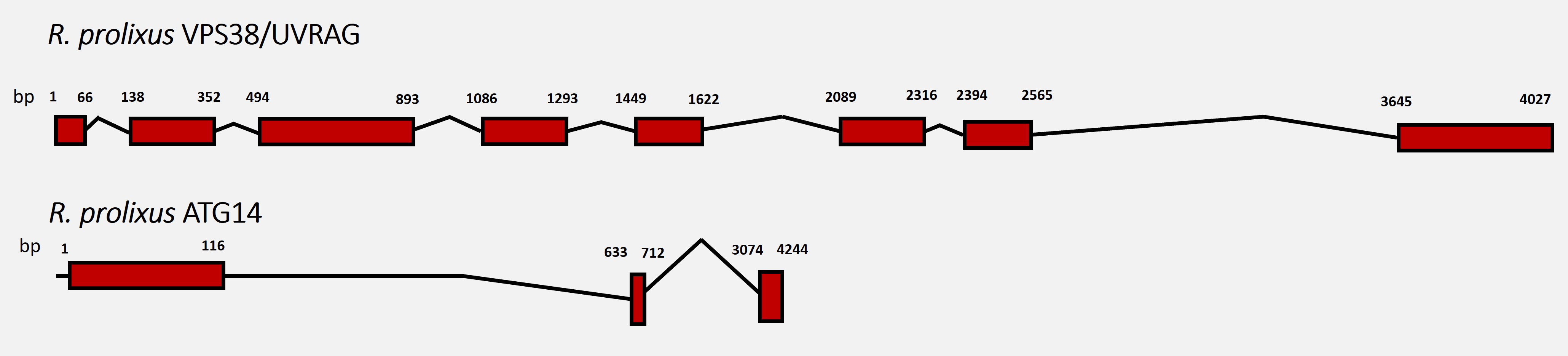

Supplement: S2 Fig — Sequence information was obtained from Vector Base (https://www.vectorbase.org/). (TIF) [file pntd.0009760.s002.tif]

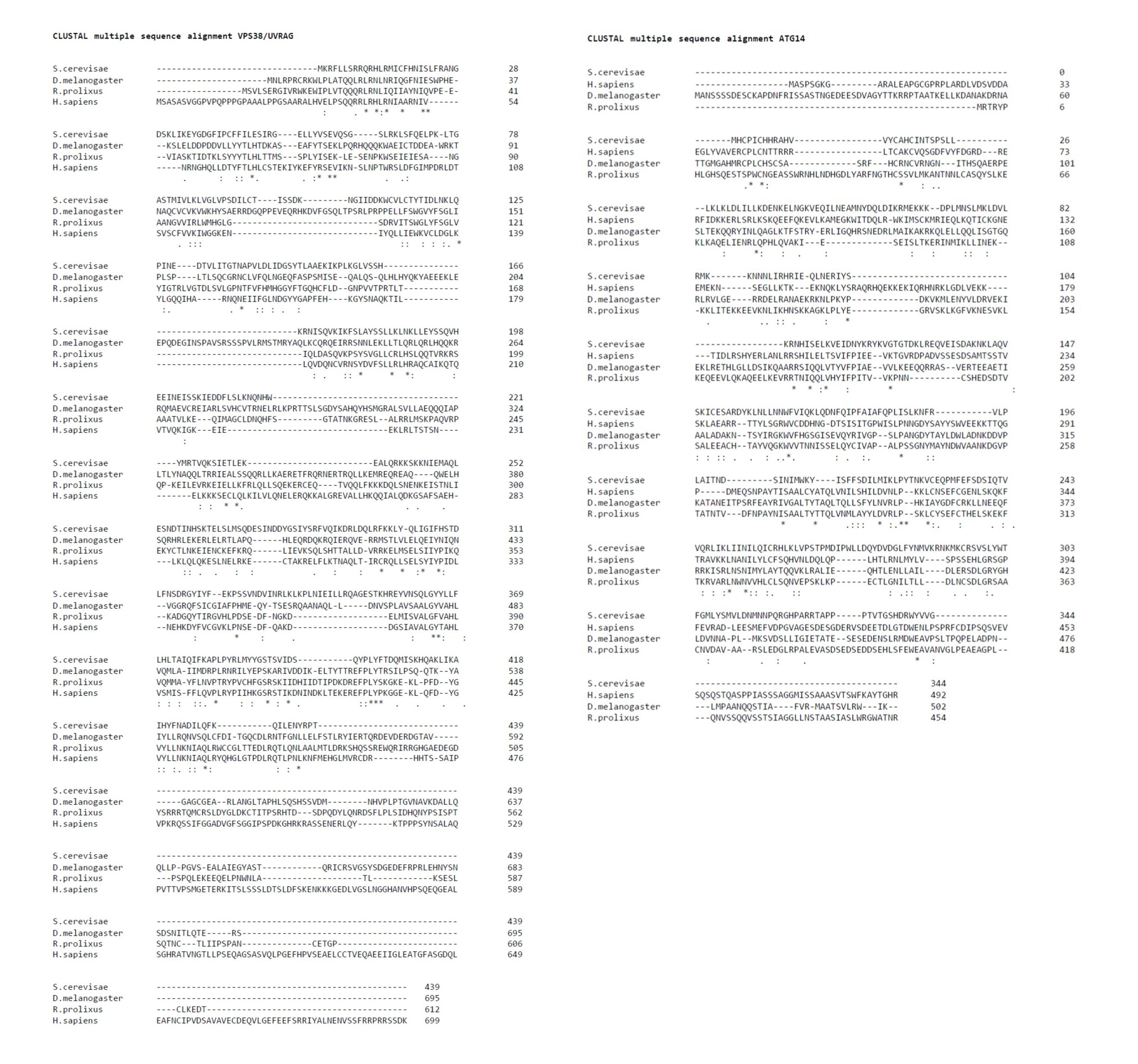

Supplement: S3 Fig — Reference sequence Rp–Rhodnius prolixus. Dm–Drosophila melanogaster; Hs–Homo sapiens; Sc—Saccharomyces cerevisiae. (TIF) [file pntd.0009760.s003.tif]

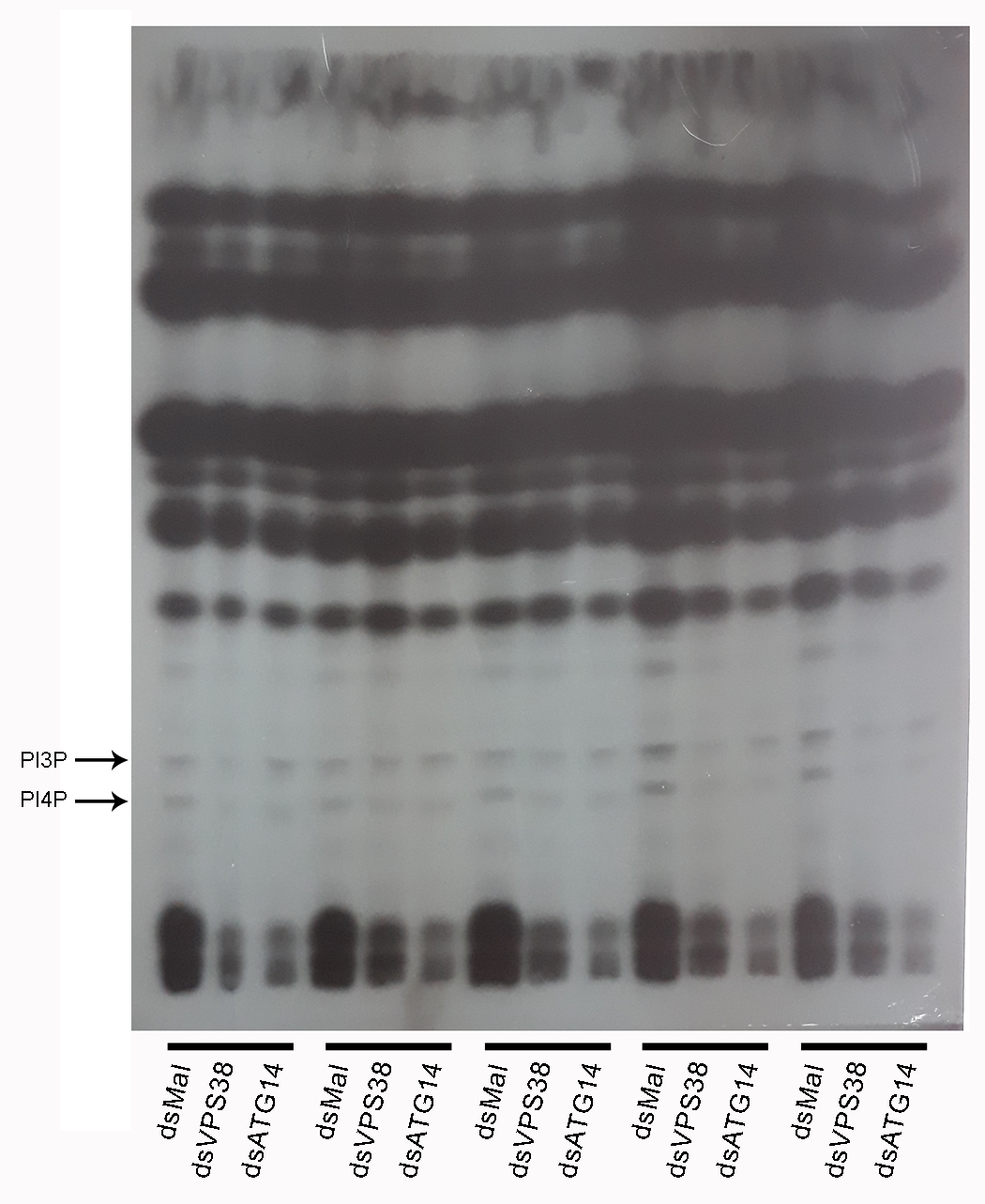

Supplement: S4 Fig — Control and silenced females were fed with a 32P-enriched blood meal. The chorionated oocytes were collected and subjected to lipid extraction followed by TLC and autoradiography. Arrows indicate the spots for PI3P and PI4P as previously described by [61]. (TIF) [file pntd.0009760.s004.tif]

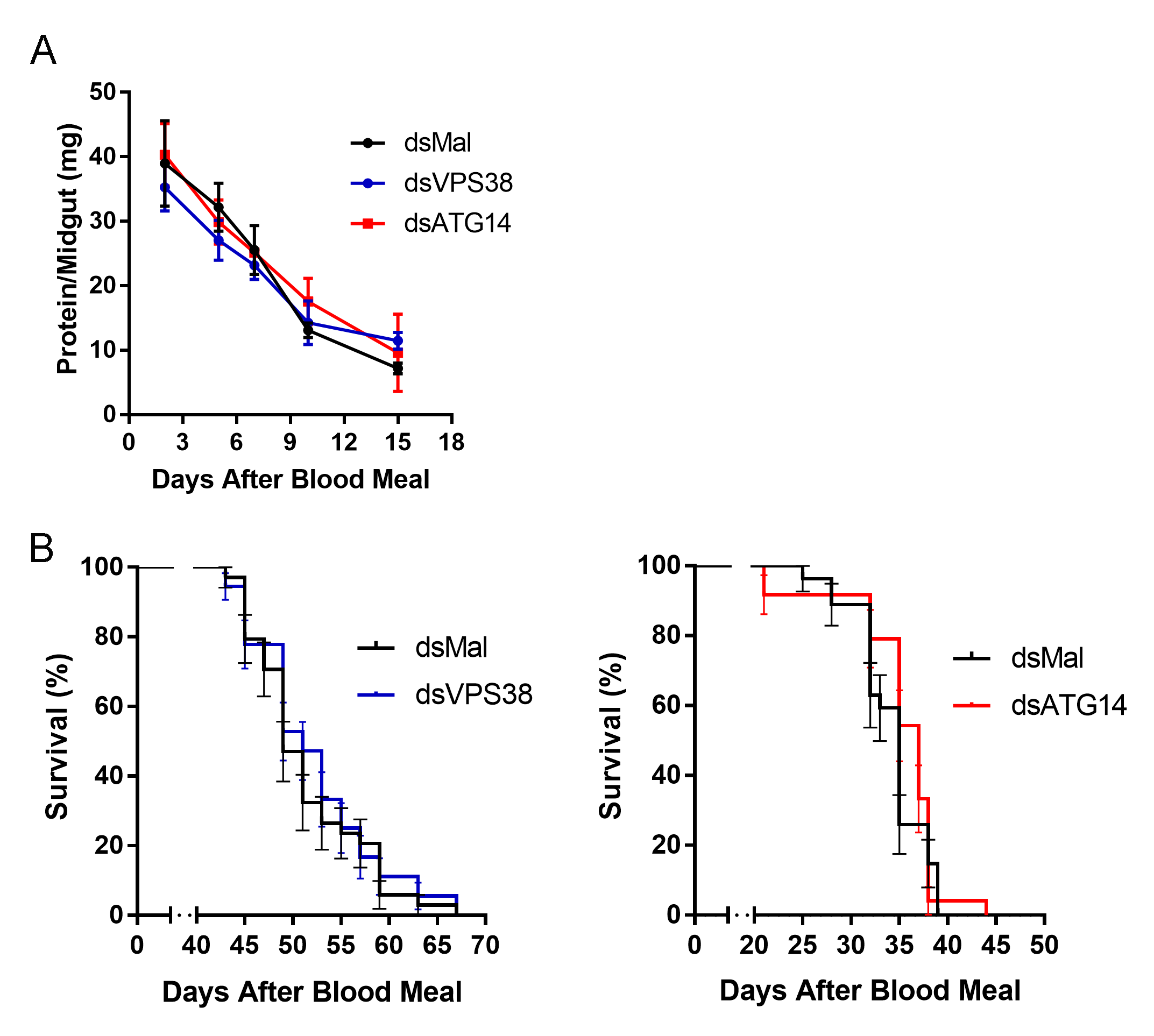

Supplement: S5 Fig — A. Effect of VPS38/UVRAG and ATG14 knockdown in the blood protein amount in the midgut of the females during digestion (n = 3). B. Survival curves of control and silenced females (n = 3). Graph shows mean ± SEM. p = 0.57 (VPS38) and p = 0.43 (ATG14), Log-rank (Mantel-Cox) test. (TIF) [file pntd.0009760.s005.tif]

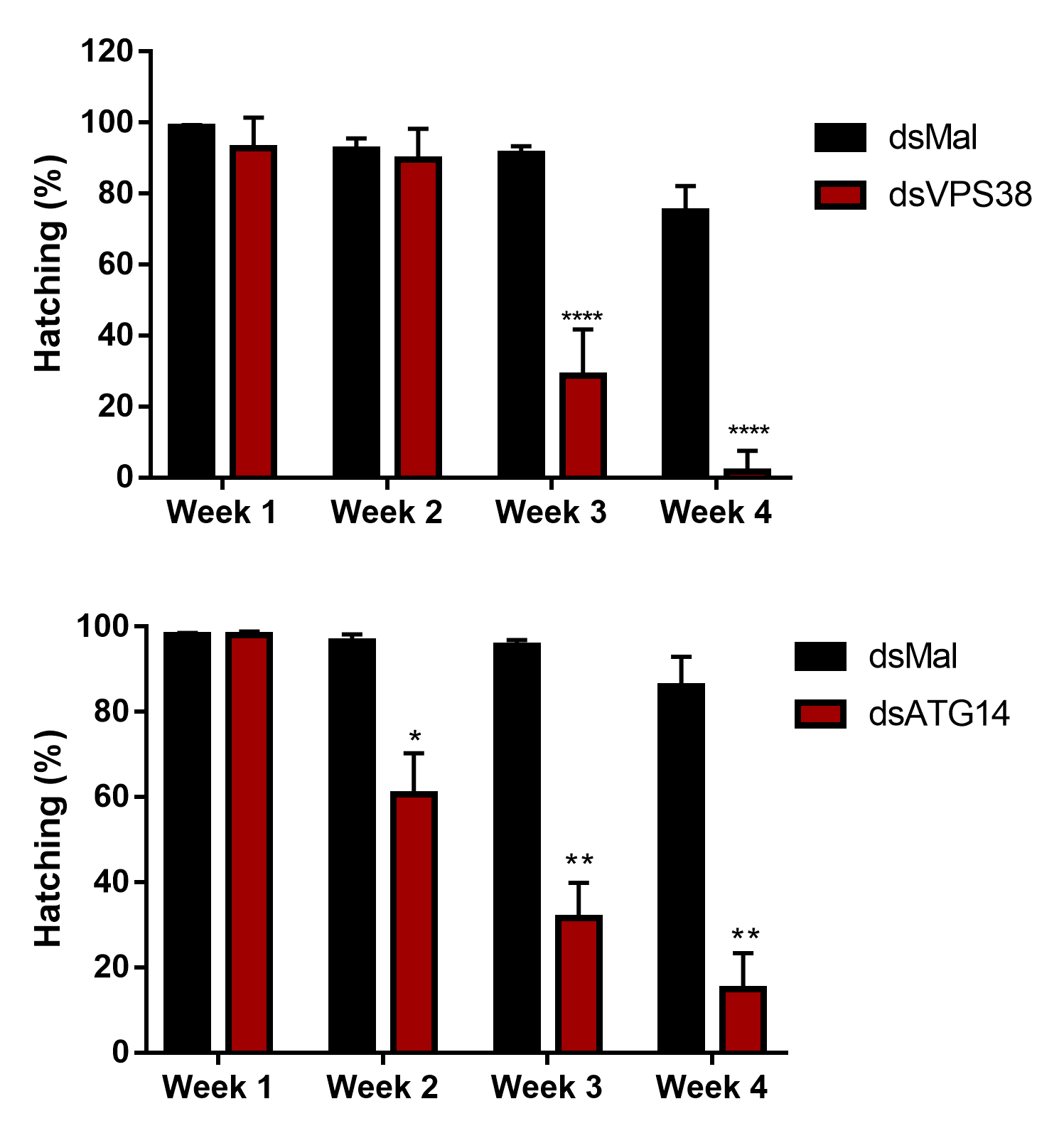

Supplement: S6 Fig — The major hatching phenotypes were observed in eggs produced during the second and third cycles of oviposition. (TIF) [file pntd.0009760.s006.tif]
