# Supplementary material for: VPS38/UVRAG and ATG14, the variant regulatory subunits of the ATG6/Beclin1-PI3K complexes, are crucial for the biogenesis of the yolk organelles and are transcriptionally regulated in the oocytes of the vector Rhodnius prolixus
Source: PLoS Negl Trop Dis. 2021 Sep 7;15(9):e0009760. doi: 10.1371/journal.pntd.0009760 (PMC8448300; doi:10.1371/journal.pntd.0009760)
Supplement: S1 Table — All sequences were obtained from Vector Base (https://www.vectorbase.org/) and primers were synthesized by Macrogen or IDT technologies. (DOCX) [file pntd.0009760.s007.docx]

| **GENE** | **VECTOR BASE** | **PRIMERS (5’-3’)** | **AMPLICON (bp)** |
| --- | --- | --- | --- |
| **ATG8/LC3** | RPRC014434-RA | FOR: GAACAATGTAATCCCACCGACAAG  REV: CCATAGACATTTTCATCACTATACGC | 306 |
| **18S** | RPRC017412-RA | FOR: TCGGCCAACAAAAGTACACA  REV: TGTCGGTGTAACTGGCATGT | 105 |
| **EF1** | RPRC007684-RA | FOR: GATTCCACTGAACCGCCTTA  REV: GCCGGGTTATATCCGATTTT | 92 |
| **PERK** | RPRC004775-RA | FOR: CGCCTGGTATCAGCACTTCTTCG  REV: CCACAGCTCTGACGATTTGATTG | 171 |
| **ATG1/ULK1** | RPRC009624-RA | FOR: GTACTGGCGTTGAGTGAATGTG  REV: CATTGCTTACTGTAGGCGATGG | 166 |
| **ATG14 (qPCR)** | RPRC001958-RA | FOR: TTGGGTTGCAGCGAACAAAG  REV: TTCAGTCTGGCTACGCGTTT | 219 |
| **ATG14 (dsRNA)** |  | FOR: ggccgcggCAGGCTTTCCCTACCCTTATTT  REV: cccggggcCAGGCTTTCCCTACCCTTATTT | 632 |
| **VPS38/UVRAG (qPCR)** | RPRC001388-RA | FOR: TTCGCCCACAGCCTAAAGAG  REV: ATCGGGAAGATGAACCCCTC | 323 |
| **VPS38/UVRAG (dsRNA)** |  | FOR: ggccgcggGTGAGGTGGAAAGAGTGGATAC  REV: cccggggCCAGGCTTTCCCTACCCTTATTT | 667 |
| **ATG6 (qPCR)** | RPRC006439-RA | FOR: CCGCTCCTGTAGACTGGTC  REV: GCCACCATCGCAGCATCAAATTTTG | 226 |
| **ATG6 (dsRNA)** |  | FOR:ggccgcggGCAGTTTGGGAGAACATACTCTCG  VER: cccggggc CTGTACACTTCTGTGTTCATCTTCC5 | 595 |
| **T7 adaptor** | - | FOR: GAGAATTCTAATACGACTCACTATAGGGCCGCGG  REV: AGGGATCCTAATACGACTCACTATAGGGCCCGGGGC | - |
