# Supplementary material for: VPS38/UVRAG and ATG14, the variant regulatory subunits of the ATG6/Beclin1-PI3K complexes, are crucial for the biogenesis of the yolk organelles and are transcriptionally regulated in the oocytes of the vector Rhodnius prolixus
Source: PLoS Negl Trop Dis. 2021 Sep 7;15(9):e0009760. doi: 10.1371/journal.pntd.0009760 (PMC8448300; doi:10.1371/journal.pntd.0009760)
Supplement: Attachment — Submitted filename: Vieira 2021, authors answers.docx [file pntd.0009760.s008.docx]

**Author’s answers (PNTD-D-21-00532)**

**Reviewer #1:**

Reviewer #1: The objectives of the study are clearly articulated with the hypothesis stated and the design is appropriate to address the stated objectives.

**Author’s Answer:** We thank the reviewer for the positive recommendation.

However, important things or clarifications must to be address to ensure reproducibility of the results and credibility in the eyes of readers, in other words, a lot of information is missing, mainly in material and methods; that section is the heart of the article because it shows the validity of the work, so please check it and be sure you lead every suggestion (explaining or adding the missing information).   

1)      Add the corresponding reference to R. prolixus genome and transcriptome databases, for example, Mesquita et al., 2015 or Giraldo-Calderón et al., 2015.

**Author’s Answer:** Done.

2)      Please, consider citing the reference to SIA software and PFAM.

**Author’s Answer:** Done. The website for SIAS and website and reference for PFAM [1] were added.

http://imed.med.ucm.es/Tools/sias.html

http://pfam.xfam.org/

3)      GenBank/database accession numbers should be provided for all of the genes/transcripts covered in the manuscript (i.e. Drosophila, yeast and Human sequences used in in Fig 1). Also, the use of Yeast and Human sequences is missing in Material and Methods.

**Author’s Answer:** We thank the reviewer for pointing it out. Gene IDs were added for all genes to the methodology>Bioinformatics.

4)      Insects section: are the insects also controlled by light and dark cycles? were the females mated? Were females in the first reproductive cycle used? In other words, after the first blood feeding as adult? What was the age of these females? Is the autogeny of these females controlled? All these questions should be answered in the manuscript to cover a rigorous methodology because the reproductive physiology in this specie, including the development of the oocytes, depends of all these parameters.

**Author’s Answer:** Agreed. The following text was added to the revised manuscript. Methodology>Insects:

“Insects were maintained at a 28 ± 2 °C controlled temperature, relative humidity of 70-80% and 12/12 h light and dark cycles. All females used in this work were obtained from our own insectarium where mated females are fed for the first time (as adult insects) in live-rabbit blood 14 to 21 days after the 5th instar nymph to adult ecdysis. After the first blood feeding, all adult insects in our insectarium are fed every 21 days. For all experiments, mated females of the second or third blood feeding were used, and dissections were carried out at different days after the blood meal depending on the experiment.”

Regarding autogeny: The concept of autogeny does not exactly apply to *R. prolixus* since it is a strict hematophagous species. The only interval when an adult female is not fed on blood is during the 21 days after the 5th instar nymph to adult ecdysis (before the first blood meal as an adult), and no eggs are laid during this period.

5)      The authors need to give more detail for the extraction methods, cDNA synthesis and qPCR (in what buffer the tissues were dissected?

**Author’s Answer:** The tissues were dissected in PBS. This info was added to methodology> Dissection of the ovary parts, follicles, and chorionated oocytes.

…detail the cDNA synthesis reaction (amount of enzyme, reaction time and temperature, the primer used), detail the qPCR reaction (amount of primers, amount of cDNA (dilutions?), temperature cycle, use of blank without cDNA, detail the characteristics of the primers (add to Table 1: slope of the curve, intercept y and r2 of the cDNA curve), how ensure that treatment with DNAse I has degraded all possible contamination with genomic DNA?) The authors might find missing information that should be added using the MIQE.

**Author’s Answer:** All of the information was added to the revised manuscript methodology>Extraction of RNA and cDNA synthesis and PCR/qPCR.

6)      For each experiment, did the authors use a pool of tissues or an individual tissue? Please, check it throughout the MS and add the information where necessary.

**Author’s Answer:** Done. The info was added to the methodology for each experiment.

7)      How were the different parts of the ovariole obtained? Perhaps it could be necessary to define the different parts of an ovariole and how they were classified in this work to be dissected. Can the authors ensure that there is no contamination between the different parts?

**Author’s Answer:** The different parts of the ovariole are carefully dissected using fine tweezers and dissecting scissors under the stereo microscope according to [2], and the structures (tropharium, previtellogenic and vitellogenic follicles and chorionated oocytes) were classified by length and morphology according to [3,4].

Although we performed the dissection carefully, contamination between tissues cannot be ruled out in most experiments where tissues/parts are dissected. This information was added to a new section in the methods of the revised manuscript intitled “Dissection of the ovary parts, follicles, and oocytes”. The revised text now reads:

“**Dissection of the ovary parts, follicles and oocytes:**

The different parts of the ovariole are carefully dissected using fine tweezers and dissecting scissors under the stereo microscope according to [2], and the structures (tropharium, previtellogenic and vitellogenic follicles, and choriogenic oocytes) were classified by length and morphology according to [3,4]…”

8)      Also, in the Supplementary Fig 1 the author stated “different tissues of the ovary and oocyte stages” but it is actually different parts of an ovariole (not tissues). Please, check it throughout the MS.

**Author’s Answer:** Checked and corrected in Fig S1.

9)      In the supplementary Fig 1, it is stated that all samples were dissected 7 days after the blood meal but in Material and Methods section, it is reported that all organs were dissected 6 days after blood meal. Please, check it. I guess at 7 days PBM the egg laying has started, but again, it depends of all the condition asked in the 4th point.

**Author’s Answer:** It was a mistake. All organs were dissected 7 days after the blood meal. For the experiments using chorionated oocytes, they were dissected 7-12 days after the blood meal (please see our answer to the comments #4 and #18).

10)     Did the authors separate follicle cells from oocytes? Otherwise, they must to be classified as follicles and not oocyte. Please, check it throughout the MS. Also, chorionated oocytes are actually eggs, right? So, how was “chorionated oocytes sample” defined if the egg laid has already started? I assume that just the eggs that are in the ovary but not in the oviduct. This is not very clear, please explain better.

**Author’s Answer:** The whole previtellogenic and vitellogenic follicles were used. This was corrected throughout the text in the revised manuscript.

Regarding the chorionated oocytes/eggs terminology:

An oocyte becomes an egg through meiotic maturation, which is the resumption of meiosis from its first arrest to reach the second arrest and become competent for activation/fertilization. For several species, the triggering hormones and morphological markers of oocyte maturation and activation are known [5–10]. In Drosophila, activation is triggered by ovulation preceding fertilization [5,9]. In *R. prolixus*, we do not know when oocyte maturation or activation occur, so there is no way to know for sure if the chorionated oocyte dissected from the ovary is an oocyte or a mature egg ready to be activated and fertilized. This is the main reason why we do no dissect chorionated oocytes from the oviduct (to avoid using them in case they are activated, as it happens in Drosophila).

Taking this information into account, we decided to maintain the chorionated oocyte terminology for the oocytes dissected from the ovary (not from the oviducts), so it matches the works in Drosophila. We added this information in the methodology>Dissection of the ovary parts, follicles, and oocytes:

The revised text now reads:

“…The chorionated oocytes were dissected from the ovarioles (not from the oviducts). Because it is not known when oocyte maturation or activation occurs in *R. prolixus*, there is no way of knowing if the chorionated oocyte is still an immature oocyte or a mature egg. The terminology chorionated oocyte (and not chorionated egg) was chosen to refer to the oocytes right after the completion of choriogenesis because it matches to the terminology used in Drosophila [5,9]…”

11)     The RNAi experiments were done lack an off-target control. Without this control, off-target effects could not be ruled out. The authors made mention of this point in the discussion, but for future work, it would be interesting to have a second dsRNA with a different sequence to exclude this possibility, specially because mostly the conclusions got by this report are based on RNAi experiments.

**Author’s Answer:** Agreed.

12)     Why does the Table S1 show two pairs of primers per each transcript to RNAi experiments? Please, explain better the RNAi design.

**Author’s Answer:** The first pair of primers were designed to amplify a 100-200 bp fragment of the transcripts and were used for qPCR detection. The second pair of primers were designed to amplify a larger 500-700 bp fragment of the transcript and contained an adaptor sequence to allow T7 in vitro transcription of the dsRNAs.

Table S1 now indicates the primers used for qPCR and dsRNA synthesis for each gene.

13)     Where is the dsRNA injected? In what part of the body insect? Was the dsRNA diluted in water, PBS or Rhodnius solution? Please, add this information to the text.

**Author’s Answer:** “Unfed adult females were injected between the second and third thoracic segments using a 10 µl Hamilton syringe with 1 µg dsRNA (diluted in 1µl of water) and fed 2 days later”. This sentence was added to the revised manuscript. Methodology>RNAi silencing.

14)     Please, specify in which tissues the knockdown efficiency was confirmed.

**Author’s Answer:** Ovary and fat body. The following sentence was added to the methodology>RNAi Silencing:

“Knockdown efficiency was confirmed by qPCR at 7 days after the blood meal in the ovary and fat body”.

15)     Figure supplementary 2: Mortality curve is not analyzed with parametric statistics since it has a binomial distribution (dead or alive). An analysis using the Log-rank test would be more appropriate.

**Author’s Answer:** It was a mistake. It was analyzed using the Log-rank (Mantel-Cox) test. We corrected the figure legend and added the p value for each curve.

16)     PI3P detection by thin-layer chromatography (TLC) and quantification: The TLC plates and the autoradiography of the phospholipids and standards used in this experiment should be show as Supplementary figure.

**Author’s Answer:** Agreed. However, there was no information on the TLC itself. Radiation was used to amplify the signal of the phospholipids and visualize them. The TLC autoradiography was added to the revised manuscript as a Supplementary Figure 4. The phospholipids migration patterns indicated were described in [11].

17)     The densitometric analysis using Adobe Photoshop CC 2015 software might not be the best option, other applications such as ImageJ are more specific for this analysis. Otherwise, please give references to use Adobe Photoshop CC 2015 software for this analysis.

**Author’s Answer:** Agreed. The densitometry was performed again using ImageJ.

18)     In Material and methods, it is stated that at 10 days post blood meal, the “chorionated oocytes” were collected; however, in the Fig 2 caption, it is stated that the organs were collected at 7 d post feeding. Also, the caption of Fig 2 indicates that PI3P detection by TLC was done using ovaries; however, in Material and methods was pointed out that just “chorionated oocytes” were used for this experiment.

**Author’s Answer:** We apologize for the confusion in the text.

For all experiments that used chorionated oocytes, they were dissected from the ovary (not from the oviduct) 7 to 12 days after the blood meal. The time range was due to the fact that different experiments (with different batches of silenced insects) produced the white/small oocytes phenotypes at slightly different times after the blood meal. The phenotype was observed as early as 7 days in some insects and as late as 12 days in others.

For the experiments where the organs were dissected, all samples were dissected at day 7 after the blood meal. The PI3P detection was performed using chorionated oocytes. The caption of Figure 2 was corrected.

19)     Egg homogenates and SDS-PAGE: were the samples centrifugated or just homogenized and used? Please, give details of the method to homogenization and the recipe for PBS.

**Author’s Answer:** Just homogenized using a plastic pestle to break the eggs in the buffer and used. The PBS recipe was added.

20)     Hemolymph extraction and SDS-PAGE: how was the hemolymph extraction done? Indicate the method used to obtain hemolymph. Was the hemolymph sample hemocyte-free? Please, indicate if the samples were centrifugated.

**Author’s Answer:** The samples were not centrifuged. The hemolymph was extracted as originally described by [12].

This info was added to the revised manuscript in Methods> Hemolymph extraction and SDS-PAGE. The revised text now reads:

“The hemolymph was extracted from control and silenced females, 7 days after the blood meal, as originally described by Masuda e Oliveira, 1985 [12]. Approximately 10 µl of hemolymph per female was obtained by cutting one of the insects’ legs and applying gentle pressure to the abdomen. The hemolymph was collected using a 10µl pipette plastic tip. Once collected, the hemolymph was diluted 2x in PBS containing protease inhibitors (aprotinin 0.3 µM, leupeptin 1 µg/µl, pepstatin 1 µg/µl, PMSF 100 µM and EDTA 1 mM), and approximately 8 mg of phenylthiourea. The equivalent of 1 µl of hemolymph was loaded in each lane of a 15 % SDS-PAGE. For each biological replicate (n=4), the samples were prepared using a pool of 3 insects”.

21)     Did the authors use around 2 X more proteins for dsVPS38 or dsATG14 than dsMaI to load SDS-gel? It is confusing because an incorrect conclusion could be drawn from this experiment, indeed, the author conclusion is that the overall levels of protein in the hemolymph were increased in knockdown insects respect to controls, but it is complicated to analyze if the protein load for each condition was different. It could be concluded that yolk protein precursors are accumulated in hemolymph if the amount of protein load is the same; otherwise you might see more yolk protein because it loaded more. Please, check it or explain better.

**Author’s Answer:** We chose to run the gel with the same volume of hemolymph from both control and silenced females (and thus different amounts of total protein) exactly because we wanted to see which proteins were accumulated in the silenced samples by observing the size of the fragments in the gel.

 22)     In Material and methods, it was written “dsRpATG6 samples”, but I think it was a mistake.

**Author’s Answer:** It was a mistake. Corrected for “dsVPS38/UVRAG and dsATG14 samples”.

23)     “Hemolymphs” should be changed by “hemolymph samples”.

**Author’s Answer:** Done.

24)     Light microscopy: were the tissues obtained only from opercula-free oocytes? Because in Material and methods only the preparation of this samples is indicated but then it is stated that transversal sections of the oocytes and eggs were obtained in a cryostat. Please, check it.

**Author’s Answer:** All samples were obtained from the opercula-free chorionated oocytes, dissected from the ovary (not from the oviduct). The text was wrong. “…and eggs” was deleted in the revised manuscript. We apologize for the mistake.

The revised text now reads:

“Once infiltrated in pure OCT, 15-30 µm transversal sections of the chorionated oocytes were obtained in a cryostat.”

What was the age of the females used for this experiment? Please, indicate this relevant information in the text.

**Author’s Answer:** The info was added to the methodology>Insects:

The revised text now reads:

“All females used in this work were obtained from our own insectarium where mated females are fed for the first time (as adult insects) in live-rabbit blood 14 to 21 days after the 5th instar nymph to adult ecdysis. After the first blood feeding, all adult insects are fed every 21 days. For all experiments, mated females of the second or third blood feeding were used, and dissections were carried out at different days after the blood meal depending on the experiment.”

How many days after blood meal?

For dissecting chorionated oocytes the females were dissected 7-12 days after the blood meal, depending on the days that the small/white phenotypes were more robust. This varied among the different experiments.

25)     Yolk organelles suspension and Flow Cytometry (FACS): The gating strategy for FACS analysis assessed by Flowing software to discriminate single yolk organelles from aggregate (doublets, clumps or debris) should be added as Supplementary Figure.

**Author’s Answer:**

Regarding the identification of aggregates/clumps:

The aim of this experiment was to provide a view of the entire population of yolk organelles obtained from chorionated oocytes in terms of size (FSC) and internal complexity (SSC), and to compare eventual changes between control and silenced samples. Isolation of the yolk organelles in *R. prolixus* have been performed before. The organelles can vary in size from 40µm to 500 nm [13,14]. For reference, please see the image below showing a typical suspension of yolk organelles obtained from a chorionated oocyte as seen by DIC microscopy.

Due to their vast variation in size, and because there is no reference or labeling available, it is not possible to differentiate between aggregates in these experiments as routinely performed in typical cell culture FACS experiments, where populations can be label-identified. However, the yolk organelles are clearly visible in the suspensions and a general comparison between the patterns of size and complexity between control and silenced samples can be accessed. Our lab is currently working on developing specific labels for different yolk organelles and we consider this technique very promising in terms of observing changes in the general population of yolk organelles isolated from oocytes.

Regarding the gating strategy:

There was no gating strategy. The aim of the experiment was to provide a view of the entire population of yolk organelles obtained from chorionated oocytes. Due to their vast variation in size, we reviewed our data and decided to perform the experiments again using different detector voltage settings in the equipment, allowing the identification of a broader range of events. The new plots are now included in the revised manuscript.

This info was added to the results of our revised manuscript. The revised text is copied below.

“To further investigate the characteristics of the yolk organelles formed in the oocytes of silenced females, we used flow cytometry to get further insight into the changes in the population of organelles in terms of size (FSC) and internal complexity (SCC), which is an unprecedent attempt. Isolation of the yolk organelles in *R. prolixus* have been performed before and the vesicles can vary in size from 500 nm to 40 µm [13,14]. Figure 5B shows representative dot-plots of the yolk organelles present in chorionated oocytes from control and silenced females. The representative plots allowed the identification of a broad range of events and showed that although the organelles are dispersed over a large range of sizes and internal complexities, the organelles from control and silenced samples presented different profile as quantified in Figure 5C. For both VPS38/UVRAG and ATG14, there is an increase in frequency of the organelles in the left bottom quadrant (LB), indicating reduced complexity and size in the general population of the yolk organelles obtained from silenced samples. Interestingly, for ATG14 the high complex and smaller organelles are decreased in frequency (left top quadrant, LT), whereas for VPS38/UVRAG the low complex and larger organelles are less frequent (right bottom quadrant, RB)”.

Also, “PBS” should be abbreviated because it has already been used.

**Author’s Answer:** Done.

What was the age of the females used for this experiment? How many days after blood meal? Please, indicate this relevant information in the text.

**Author’s Answer:** For all experiments females from the second or third blood meal were used (explained in the revised manuscript Methodology>Insects). For obtaining the chorionated oocytes the females were dissected 7-12 days after the blood meal, depending on the days that the small/white phenotypes were more robust. This varied among the different experiments.

26)     Please clarify in Material and Methods section the number of independent experiments, and the number of samples (individual or pools) composing each experimental group. This is valid for all the experiments described in the work.

**Author’s Answer:** Done. Our apologies. The biological replicates (n) and the info about pools or individual samples were added to the methodology for each experiment.

Reviewer #1: Results.

1) Results: please, add as Supplementary information the alignment between the different sequences in order to demonstrate the percentage of similarity indicated in “Results”, as well as a picture showing the exons indicated by the authors for each transcript.

**Author’s Answer:** As suggested, the images were added as Figures S2 and S3 in the revised manuscript.

2)      Figure 1: indicate the statistical significance of the asterisk in the caption.

**Author’s Answer:** Done.

3)      The VPS38 and ATG14 transcript expression showed in Figure 1 is so different to that reported in Figure 2 (control insects). Please, explain this point.

**Author’s Answer:** We sincerely apologize for this mistake and thank the reviewer for pointing it out. Although the relative pattern of expression was correct in Figure 1D, the numbers presented were miscalculated. We have performed the experiments again and added the corrected graph to the revised manuscript.

4)      In Figure 2 A-B: is the complete ovary used for this experiment?

**Author’s Answer:** Yes.

5)      Figure 2 C: please, be careful to discuss about if the complete ovary or just the chorionated oocytes have been used, it should not be used indistinctly. This is valid for all the manuscript.

**Author’s Answer:** Agreed. The samples are now better described throughout the revised manuscript.

6)      Figure 2 C: It is not clear that silenced ovaries are presenting a reduction of 72% in their PI3P levels, it seems closer to 40%.

**Author’s Answer:** Corrected. The relative densitometric reduction was calculated from an average of 70.9 (dsMal) to approximately 29.6 (dsVPS38 and dsATG14). A reduction of approximately 60%.

7)      Fig 3E is not in Figure 3, please, check the text in results section.

**Author’s Answer:** Corrected to Fig 3C.

8)      It can’t be concluded from a simple SDS-gel that the bands noted as Vt/Vg and RHBP are exactly what the authors claim to be. We all know the molecular weights of them, but it would be necessary to indicate it with more care if you don’t use a specific antibody. Please, write in potential (the band is compatible with …) when indicating that these proteins could be Vt/Vg and RHBP, and refer to some other paper where the running profile by SDS-gel has been demonstrated for each protein.

**Author’s Answer:** Agreed. We have performed a new SDS-PAGE (added to the revised manuscript) which allowed us to better identify the fragments in accordance with the literature. The revised text now reads as follows and the references for the fragments compatible to VT and RHBP were added.

“…and the fragments compatible with the major yolk proteins Vitellogenin [12] (Vg) (arrowheads) and RHBP [15,16] were accumulated in the hemolymph (Fig 3D).”

9)      The model for the transcriptional regulations between ATG6/Beclin1, VPS38/UVRAG and ATG14 is in Figure 6 but in the caption of Figure 5. Please, check it.

**Author’s Answer:** Corrected.

10)     Please, add the statistical significance missing in the caption of Figure 6.

**Author’s Answer:** Done.

11)     Please, clarify if the level of knockdown presented (Knockdown (%) post-treatment) is lowering the expression by presented % or to presented %. Authors could add it in the caption.

**Author’s Answer:** The text was altered in the revised manuscript for clarification. It now reads as follows:

Results:

“The VPS38/UVRAG expression levels were reduced by 70% in the ovary and by 50% in the fat body (Fig 2A), whereas the ATG14 expression levels were decreased by 80% in the ovary and by 90% in the fat body (Fig 2B).”

Fig 2 caption:

“The expression levels of VPS38/UVRAG were reduced by 70% in the ovary and by 50% in the fat body. The expression levels of ATG14 were reduced by 80% in the ovary and by 90% in the fat body.”

12)     Flow Cytometry (FACS): Is there any reference for this experiment? Otherwise, the authors should explain better what is the aim of measure the complexity of yolk organelles, because it is clear the reason to measure the forward direction (to indicate the relative size of the organelles), but the SSC is use to indicate the internal complexity or granularity of the cell, and here just yolk organelles are analyzed. Could the protein contained in each organelle (after discrimination single organelle from aggregates) give information about the complexity?

**Author’s Answer:** “It is possible that yolk organelles differences in contents may be detectable as changes in internal complexity. For example, the yolk proteins in Xenopus are packed in some yolk organelles in a crystalline structure [17], and internal vesicles and membranes have been observed inside some specific organelles in the oocytes of chicken and echinoderms [18,19]. Because we do not know much about the nature of the distinct yolk organelles in *R. prolixus*, observing their overall pattern of relative size and internal complexity showed that indeed those organelles are diverse, and most importantly for this work, this pattern is altered in the oocytes produced by silenced females. There is no reference for this experiment, and our lab intends to further explore this methodology and develop labels to detect changes in specific yolk organelles under different conditions”.

The above text was included in the discussion of the revised manuscript.

13)     Figure 5: The Fig 5B doesn’t seem a representative density plots of the yolk organelles present in control and silenced chorionated oocytes if it is compared with the frequency showed on the upper quadrants (right and left, Fig 5C). In the Fig 5B it seems that in the RT, events in dsVPS38 RT are higher than control, and in the LT, events in both dsVPS38 and dsATG14 LT, are higher than control.

**Author’s Answer:** The plot was changed for a more representative one of the quadrants frequency quantifications.

14)     Figure 6: please, add in Material and methods section the use of dsATG6.

**Author’s Answer:** The use of dsATG6 was added to methodology>RNAi silencing: The revised text includes the sentence below:

“dsRNA for the silencing of ATG6/Beclin1 was synthesized as previously described [20].”

Reviewer #1: Discussion.

1) Discussion: it seems that a word is missing in the first sentence. Please, check it.

**Author’s Answer:** Corrected.

2)      Yolk protein precursors are accumulated in coated vesicles and then, after loosing clathrin coats, in early endosomes. These early endosomes then fuse and form the yolk organelles; in this context, it would be great to discuss what of these potential levels were affected.

**Author’s Answer:** Agreed. The following text was added to the discussion of the revised manuscript:

“The participation of endocytosis in the yolk uptake is not disputed and have been previously described in the oocytes of other insects [21–29]. Because PI3P is typically highly concentrated in early endosomes [30], and the mature yolk organelles are notably not formed in the oocytes of silenced females (Fig 5), it is likely that the silencing of the ATG6-PI3K complexes impair the endocytic flux at early steps of endosomal sorting. However, the identification of the different endocytic stages that could be compromised in the oocytes of silenced insects remains to be investigated”.

3)      The fact that the expression of ATG14 is not altered by downregulation of VPS38/UVRAG and ATG6/Beclin1 allows to conclude that the impaired yolk uptake phenotype by dsATG6 treated females is the effect of silencing the VPS38 PI3K complex II, which could indicate the VPS38/UVRAG PI3K complex II as the major contributor just for yolk endocytosis, and then, could ATG14 (complex I) be working on further steps, e.g. during the process of protein degradation (autophagy)?

**Author’s Answer:** It is possible. This info was added to the discussion of the revised manuscript. Text copied below.

“It is possible that the ATG14 PI3K complex is maternally loaded during oogenesis but activated only at later processes of oogenesis or early development such as egg activation, fertilization, and early yolk degradation. Activation of the autophagic flux after fertilization has been shown in mice, where it was associated with mRNA degradation for the maternal-zygotic transition [31]. In *C. elegans*, paternal mitochondria are degraded via autophagy after fertilization [32], and, in Drosophila, ATG mutants have varied phenotypes presenting compromised embryogenesis [33]. Yolk degradation, specifically, was associated with autophagic mechanisms in 2015 in Drosophila. In this work, the authors showed that target of rapamycin (TOR) and ATG1 are important for the yolk catabolism and for the formation of autophagosomes [34]”.

Reviewer #1: Introduction:

1)      Since the scope of PLOS Neglected Tropical Diseases is aimed to report studies showing aspects of forgotten diseases affecting the world’s most neglected people, information about the relevance of this work in the current context of Chagas disease should be added, specially because since 2005, the WHO recognized this affection as a neglected tropical disease (NTDs), which is mainly transmitted by the contact of people with faeces/urine of infected blood-sucking triatomine bugs.

**Author’s Answer:** Agreed.

The following text was added to Discussion in the revised manuscript:

“In 2005, the WHO created the department of Neglected Tropical Diseases (NTDs), recognizing their importance and aiming to manage their incidence mostly in Africa and Latin America (<http://www.who.int/neglected_diseases/en/>). *Rhodnius prolixus* is one of the main vectors of Chagas disease, which is one of the several NTDs that are important in Latin America. Currently 6 to 7 million people are estimated to be infected by Chagas Disease, and vector control is still the most useful method to prevent this illness. Because embryo development relies entirely on the buildup of the yolk nutritional reserves, the precise biogenesis of the yolk organelles is indispensable for vector reproduction representing a promising target for interference in vector’s reproduction.”

2)      Also, I realized that a good background talking about endocytic organelles in insect oocytes, or showing the nature of yolk bodies, was missing; it should be added in the introduction to show the relevance of the complexes studied here, in the regulation of autophagy and membrane dynamic of endocytosis.

**Author’s Answer:** Agreed. The following text was added to the introduction of the revised manuscript:

“The participation of receptor mediated endocytosis in the yolk uptake and biogenesis of the yolk organelles have been investigated in oocytes of many species. In insects, the internalization of yolk proteins through the presence of a specialized endocytic cortex in the oocytes, which includes prominent microvilli, coated pits, coated vesicles, and endosomes have been shown in in several species [21–28]. Immunodetection of the internalization of yolk proteins and markers of endocytosis (such as clathrin, α-adaptin, and yolk protein receptors) have been performed in Drosophila [28] and mosquito [29]. However, the regulations encompassing the recruitment of the endocytic machinery to the specific sites of the oocyte cortex and the signals and regulations that govern the oocyte endocytic pathways and endosomal maturation have never been addressed.”

3)      Could the authors check the references, and perhaps use more reviews when possible? for example, at the end of a sentence in the introduction there are 14 references added; I understand that it is also part of previous stated, but in that case, it would be better to use after each sentence the corresponding reference.

**Author’s Answer:** Done.

4)      ATG9 is named in the 3rd paragraph, I suggest that each time something new is named in the text, it is briefly defined.

**Author’s Answer:** Done.

5)      It may be helpful for the reader to indicate the full name of the abbreviation the first time they are named, for example, UV radiation resistance-associated gene protein (UVRAG) Vacuolar protein sorting 34 (Vps34), AuTophaGy-related (ATG), etc.

**Author’s Answer:** Done.

Reviewer #1: In this study, the authors performed an analysis on the biogenesis of yolk organelles in the oocytes of the vector Rhodnius prolixus, delving into a study previously published by the same group. Here they report that VPS38/UVRAG and ATG14 subunits of the ATG6/Beclin1-PI3K complexes are essential for the yolk endocytosis. The work is original in the study area on biogenesis of yolk bodies and the conclusions are justified by the evidence presented. Overall, I consider it work contains very interesting and relevant data and deserves to be published. However, it is essential to address a significant number of points to improve the understanding and reproducibility of the experiments. Also, I want to state that the page numbers and line numbers in the manuscript file were missing, so it has been a bit complicated to indicate my observations.

**Author’s Answer:** We thank the reviewer for the positive recommendation. All points were answered. Page and line numbers were added.

**Reviewer #2**

Reviewer #2: The objective and hypothesis are well articulated. In general terms, the experimental designs are appropriate. Sample size is adequate to address the hypothesis. Statistical analyses were correctly applied.

**Author’s Answer:** We thank the reviewer for the positive recommendation.

Reviewer #2: The analysis presented are in agreement with the analysis plan. The results are completely presented. Figures and Tables are of sufficient quality. Minor revision for specific Figures was indicated in "comments to authors".

**Author’s Answer:** We thank the reviewer for the positive recommendation.

Reviewer #2: Most of conclusions are supported by the findings, but discussion needs to be improved. Please, see “comments to authors” for details.

Reviewer #2: Comments to the authors:

In this work, Veira and co-authors analyzed the role of the variant subunits VPS38/UVRAG and ATG14 in the biogenesis of the yolk bodies in the insect vector of Chagas Disease Rhodnius prolixus. Both genes are members of the ATG6/Beclin1 class-III PI3K complexes, which are involved in the regulation of autophagy and the membrane dynamics of endocytosis. The aims are clear and straightforward, and the results are compelling. Even though some conclusions must be revised, the manuscript open horizons to unravel the regulating processes involved in protein uptake and trafficking by developing oocytes, which are largely unknown. Please, find below some points that I suggest revising in order to improve this manuscript.
**Author’s Answer:** We thank the reviewer for the positive recommendation.

General Comments:

- Through the manuscript: Sentences like “control and silenced eggs …” or “chorionated oocytes..” are incorrect.  Samples were obtained from control and silenced females. Please, revise.

**Author’s Answer:** Done.

- Material and Methods, page 10- Yolk organelles suspension and Flow Cytometry (FACS): Consider replacing “Suspensions of yolk organelles were obtained by gently disrupting recently dissected chorionated…” by “Suspensions of yolk organelles were obtained by gently disrupting of recently dissected chorionated…”

**Author’s Answer:** Done.

- Results, page 10-Silencing of VPS38/UVRAG and ATG14 results in the accumulation of yolk proteins in the hemolymph and formation of small yolk-deficient oocytes: Consider replacing “Double stranded RNAs were designed to target specific regions of the VPS38/UVRAG and ATG14 mRNAs and injected to the insects hemocoel 2 days before the blood meal” by “Double stranded RNAs designed to target specific regions of the VPS38/UVRAG and ATG14 mRNAs were injected to the insects hemocoel 2 days before the blood meal.

**Author’s Answer:** Done.

- Results, page 11- VPS38/UVRAG and ATG14 silenced eggs are yolk-deficient and do not support embryo development: Consider replacing “… the silenced females laid the same number of eggs as control females”…by “… the silenced females laid similar number of eggs as control females”

**Author’s Answer:** Done.

Specific Comments:

a) Results and Figures:

- Silencing of VPS38/UVRAG and ATG14 results in the accumulation of yolk proteins in the hemolymph and formation of small yolk-deficient oocytes (page 11): The authors proposed the measurement of hemolymph yolk protein content in control and silenced females to demonstrate that formation of white/small oocytes was due to a deficiency in the uptake of yolk proteins from the hemolymph. Such an assumption is reasonable but not conclusive if information regarding expression (mRNA and protein levels) of yolk protein precursors such as vitellogenin in the fat body is not provided. In addition, the results showed the amounts of total proteins in the hemolymph but not yolk protein content. Authors should perform at least western blots for vitellogenin and RHBP in fat body to better support the conclusion. Please, limit the rationale of the experimental design.

**Author’s Answer:** Agreed, and we already have this data. We addressed this issue in our previous paper where ATG6/Beclin1 was silenced [20]. Using HPLC and referenced standards we observed that the fat bodies from control and silenced samples secrete comparable amounts of RHBP and Vg (using in vitro secretion assays), and that the hemolymph of silenced females accumulates twice more RHBP and Vg than the hemolymph of control insects. Because both VPS38/UVRAG and ATG14 silenced insects are also silenced for ATG6/Beclin1 (as we found in this manuscript), there is no way perform this test separately in VPS38/UVRAG and ATG14 silenced insects.

Unfortunately, we do not have reliable antibodies against RHBP or vitellogenin.

-VPS38/UVRAG, ATG14 and ATG6/Beclin1 (page 12) and Figure 6: Authors should provide a hypothesis to explain the downregulation of ULK1/ATG1 in VPS38/UVRAG-silenced samples.

**Author’s Answer:** The following text was added to the discussion of the revised manuscript.

“Interestingly, however, the silencing of VPS38/UVRAG triggered the downregulation of ULK1/ATG1, and this did not occur in ATG14 or ATG6/Beclin1 silenced females, where VPS38/UVRAG was also cross-silenced. Because the crosstalk between endocytosis and autophagy is known and described in different models, it is possible that the signals for endocytosis also co-regulate the biogenesis of autophagosomes through the transcriptional regulation of ULK1/ATG1. The absence of the same effect in the samples where VPS38 was cross silenced (dsATG6/Beclin1 and dsATG14) might be due to the fact that gene regulatory networks are wired in a temporal regulated manner, so that the timing of the transcriptional downregulation was not enough to trigger the effect on ULK1/ATG1 observed when VPS38 itself was silenced. Experiments to test the levels of all three transcripts and the lipidation of ATG8/LC3 (as a readout of autophagosome formation) throughout the vitellogenesis period could help illuminate this hypothesis.

”

- Fig. 3D and Fig. 4F: Did the authors use a loading control for SDS-PAGE? According to Material and Methods section, the amount of proteins loaded were different in samples from control and silenced females.

**Author’s Answer:** We did not use loading controls because we chose to run the gel with the same volume of hemolymph from both control and silenced females (and thus different amounts of total protein) exactly because we wanted to see which proteins were accumulated in the silenced samples by observing the size of the bands in the gel.

- Legends to Figure 5 and Figure 6 (page 18): The model for the transcriptional regulations between ATG6/Beclin1, VPS38/UVRAG and ATG14 is shown in Figure 6, not in Figure 5. Please, revise.

**Author’s Answer:** Done.

- Supplementary Figure S2: Survival curves are presented in the panel “B”, but the legend stated “E-F”. Please, revise.

**Author’s Answer:** Corrected.

b) Discussion:

- Authors associated the decrease of PI3P in ovaries/chorionated oocytes from VPS38/UVRAG and ATG14 silenced females with “at least some reduction of their protein activity, validating the specificity of the observed phenotypes”. In fact, signaling mediated by PI3P is complex and can be performed even with low levels of PI3P. Please, tone down the conclusion.

**Author’s Answer:** Agreed. “…validating the specificity of the observed phenotypes.” was deleted.

- Authors scarcely compare their results with others in the literature from different species. Please complete.

**Author’s Answer:** Done. The following text was added to our discussion.

“The critical roles of the ATG6/Beclin1-associated complexes in the endocytic trafficking and autophagy pathways have been described before in models such as Drosophila and *C. elegans* [35–38]. Interestingly, in the hard tick *Haemaphysalis longicornis* depletion of ATG6/Beclin1 also results in a repression of Vg uptake by the oocytes [39] and in *Caenorhabditis elegans*, disruption of ATG6/Beclin1 results in defects in the secretion and localization of the lipoprotein receptor-related protein 1 (LRP1) [40]. Both LRP1 and Vg are important for oogenesis and their disruption might also be the result impaired endocytic activities in those models”.

1.	Mistry J, Chuguransky S, Williams L, Qureshi M, Salazar GA, Sonnhammer ELL, et al. Pfam: The protein families database in 2021. Nucleic Acids Res. 2021;49: D412–D419. doi:10.1093/nar/gkaa913

2.	Huebner E, Injeyan H. Patency of the follicular epithelium in Rhodnius prolixus: a reexamination of the hormone response and technique refinement. Can J Zool. 1980;58: 1617–1625.

3.	Bjornsson CS, Huebner E. Extracellular H+ dynamics during oogenesis in Rhodnius prolixus ovarioles. J Exp Biol. 2004;207: 2835–2844. Available: http://www.ncbi.nlm.nih.gov/entrez/query.fcgi?cmd=Retrieve&db=PubMed&dopt=Citation&list_uids=15235012

4.	Nunes-da-fonseca R, Berni M, Pane A, Araujo HM. Rhodnius prolixus : From classical physiology to modern developmental biology. 2017; 1–11. doi:10.1002/dvg.22995

5.	Heifetz Y, Yu J, Wolfner MF. Ovulation triggers activation of Drosophila oocytes. Dev Biol. 2001;234: 416–424. doi:10.1006/dbio.2001.0246

6.	Gibbs JN. Oak wilt. Arboric J. 1978;3: 351–356. doi:10.1080/03071375.1978.10590543

7.	Duncan FE, Schindler K, Schultz RM, Blengini CS, Stein P, Stricker SA, et al. Unscrambling the oocyte and the egg: Clarifying terminology of the female gamete in mammals. Mol Hum Reprod. 2020;26: 797–800. doi:10.1093/molehr/gaaa066

8.	Whitaker M. Calcium at fertilization and in early development. Physiol Rev. 2006;86: 25–88. doi:10.1152/physrev.00023.2005

9.	Horner VL, Wolfner MF. Mechanical stimulation by osmotic and hydrostatic pressure activates Drosophila oocytes in vitro in a calcium-dependent manner. Dev Biol. 2008;316: 100–109. Available: http://www.ncbi.nlm.nih.gov/entrez/query.fcgi?cmd=Retrieve&db=PubMed&dopt=Citation&list_uids=18304524

10.	Page AW, Orr-Weaver TL. Stopping and starting the meiotic cell cycle. Curr Opin Genet Dev. 1997;7: 23–31. Available: http://www.ncbi.nlm.nih.gov/entrez/query.fcgi?cmd=Retrieve&db=PubMed&dopt=Citation&list_uids=9024631

11.	Walsh JP, Caldwell KK, Majerus PW. Formation of phosphatidylinositol 3-phosphate by isomerization from phosphatidylinositol 4-phosphate. Proc Natl Acad Sci U S A. 1991;88: 9184–9187. doi:10.1073/pnas.88.20.9184

12.	Masuda H, Oliveira PL. Characterization of vitellin and vitellogenin of Rhodnius prolixus. identification of phosphorilated compounds in the molecule . Insect Bichemistry Mol Biol. 1985;15: 543–550.

13.	Ramos IB, Miranda K, de Souza W, Oliveira DMP, Lima a PC a, Sorgine MHF, et al. Calcium-regulated fusion of yolk granules is important for yolk degradation during early embryogenesis of Rhodnius prolixus Stahl. J Exp Biol. 2007;210: 138–48. doi:10.1242/jeb.02652

14.	Ramos I, Gomes F, Koeller CM, Saito K, Heise N, Masuda H, et al. Acidocalcisomes as calcium- and polyphosphate-storage compartments during embryogenesis of the insect Rhodnius prolixus Stahl. PLoS One. 2011;6: e27276. doi:10.1371/journal.pone.0027276

15.	Oliveira PL, Kawooya JK, Ribeiro JM, Meyer T, Poorman R, Alves EW, et al. A heme-binding protein from hemolymph and oocytes of the blood-sucking insect, Rhodnius prolixus. Isolation and characterization. J Biol Chem. 1995;270: 10897–10901.

16.	Machado EA, Oliveira PL, Moreira MF, de Souza W, Masuda H. Uptake of Rhodnius heme-binding protein (RHBP) by the ovary of Rhodnius prolixus. Arch Insect Biochem Physiol. 1998;39: 133–143.

17.	Karasaki, S; Komoda T. Electron Micrographs of a crystalline lattice Structure in Yolk Platelets of the Amphibian Embryo. Nature. 1958;4506: 407–408.

18.	Whalley T, Grossmann JG, Wess TJ. An analysis of the lamellar structure of sea urchin egg cortical granules using X-ray scattering. FEBS Lett. 2000;482: 242–246. doi:10.1016/s0014-5793(00)02068-8

19.	Ramos IB, Miranda K, Ulrich P, Ingram P, LeFurgey A, Machado E a, et al. Calcium- and polyphosphate-containing acidocalcisomes in chicken egg yolk. Biol Cell. 2010;102: 421–34. doi:10.1042/BC20100011

20.	Vieira PH, Bomfim L, Atella GC, Masuda H, Ramos I. Silencing of RpATG6 impaired the yolk accumulation and the biogenesis of the yolk organelles in the insect vector R. prolixus. PLoS Negl Trop Dis. 2018;12: 1–19. doi:10.1371/journal.pntd.0006507

21.	Raikhel AS, Dhadialla TS. Accumulation of yolk proteins in insect oocytes. Annu Rev Entomol. 1992;37: 217–251. doi:10.1146/annurev.en.37.010192.001245

22.	Roy S, Saha TT, Zou Z, Raikhel AS. Regulatory Pathways Controlling Female Insect Reproduction. Annu Rev Entomol. 2018;63: 489–511. doi:10.1146/annurev-ento-020117-043258

23.	Anderson KL, Woodruff RI. A gap junctionally transmitted epithelial cell signal regulates endocytic yolk uptake in Oncopeltus fasciatus. Dev Biol. 2001;239: 68–78. doi:10.1006/dbio.2001.0433

24.	Postlethwait JH, Giorgi F. Vitellogenesis in insects. Dev Biol (N Y 1985). 1985;1: 85–126.

25.	Raikhel AS, Dhadialla TS, Cho W, Hays AR, Koller CN. Aedes aegypti,. 1990.

26.	Brooks RA, Woodruff RI. Calmodulin transmitted through gap junctions stimulates endocytic incorporation of yolk precursors in insect oocytes. Dev Biol. 2004;271: 339–349.

27.	Ferenz H-J. Receptor-Mediated Endocytosis of Insect Yolk Proteins. In: Hagedorn HH, Hildebrand JG, Kidwell MG, Law JH, editors. Molecular Insect Science. Boston, MA: Springer US; 1990. pp. 131–138. doi:10.1007/978-1-4899-3668-4_16

28.	Richard DS, Gilbert M, Crum B, Hollinshead DM, Schelble S, Scheswohl D. Yolk protein endocytosis by oocytes in Drosophila melanogaster: Immunofluorescent localization of clathrin, adaptin and the yolk protein receptor. J Insect Physiol. 2001;47: 715–723. doi:10.1016/S0022-1910(00)00165-7

29.	Raikhel AS, Dhadialla TS, Cho W-L, Hays AR, Koller CN. Biosynthesis and Endocytosis of Yolk Proteins in the Mosquito. In: Hagedorn HH, Hildebrand JG, Kidwell MG, Law JH, editors. Molecular Insect Science. Boston, MA: Springer US; 1990. pp. 147–154. doi:10.1007/978-1-4899-3668-4_18

30.	Naslavsky N, Caplan S. The enigmatic endosome - Sorting the ins and outs of endocytic trafficking. J Cell Sci. 2018;131. doi:10.1242/jcs.216499

31.	Yamamoto A, Mizushima N, Tsukamoto S. Fertilization-Induced Autophagy in Mouse Embryos is Independent of mTORC11. Biol Reprod. 2014;91: 1–7. doi:10.1095/biolreprod.113.115816

32.	Al Rawi S, Louvet-Vallée S, Djeddi A, Sachse M, Culetto E, Hajjar C, et al. Postfertilization autophagy of sperm organelles prevents paternal mitochondrial DNA transmission. Science (80- ). 2011;334: 1144–1147. doi:10.1126/science.1211878

33.	McPhee CK, Baehrecke EH. Autophagy in Drosophila melanogaster Christina. Biochim Biophys Acta. 2010;1793: 1452–1460. doi:10.1016/j.bbamcr.2009.02.009.Autophagy

34.	Kuhn H, Sopko R, Coughlin M, Perrimon N, Mitchison T. The Atg1-Tor pathway regulates yolk catabolism in *Drosophila* embryos. Development. 2015;142: 3869–3878. doi:10.1242/dev.125419

35.	Juhász G, Hill JH, Yan Y, Sass M, Baehrecke EH, Backer JM, et al. The class III PI(3)K Vps34 promotes autophagy and endocytosis but not TOR signaling in Drosophila. J Cell Biol. 2008;181: 655–666. doi:10.1083/jcb.200712051

36.	Ruck A, Attonito J, Garces KT, Núnez L, Palmisano NJ, Rubel Z, et al. The Atg6/Vps30/Beclin 1 ortholog BEC-1 mediates endocytic retrograde transport in addition to autophagy in C. elegans. Autophagy. 2011;7: 386–400. doi:10.4161/auto.7.4.14391

37.	Shravage B V., Hill JH, Powers CM, Wu L, Baehrecke EH. Atg6 is required for multiple vesicle trafficking pathways and hematopoiesis in Drosophila. Development. 2013;140: 1321–1329. doi:10.1242/dev.089490

38.	Fischer JA, Eun SH, Doolan BT. Endocytosis, endosome trafficking, and the regulation of Drosophila development. Annu Rev Cell Dev Biol. 2006;22: 181–206. doi:10.1146/annurev.cellbio.22.010605.093205

39.	Kawano S, Umemiya-Shirafuji R, Boldbaatar D, Matsuoka K, Tanaka T, Fujisaki K. Cloning and characterization of the autophagy-related gene 6 from the hard tick, Haemaphysalis longicornis. Parasitol Res. 2011;109: 1341–1349. doi:10.1007/s00436-011-2429-x

40.	Roudier N, Lefebvre C, Legouis R. CeVPS-27 is an endosomal protein required for the molting and the endocytic trafficking of the low-density lipoprotein receptor-related protein 1 in Caenorhabditis elegans. Traffic. 2005;6: 695–705. doi:10.1111/j.1600-0854.2005.00309.x
